# The BiSearch web server

**DOI:** 10.1186/1471-2105-7-431

**Published:** 2006-10-05

**Authors:** Tamás Arányi, András Váradi, István Simon, Gábor E Tusnády

**Affiliations:** 1 lnstitute of Enzymology, BRC, HAS, H-1113 Karolina út 29, Budapest, Hungary

## Abstract

**Background:**

A large number of PCR primer-design softwares are available online. However, only very few of them can be used for the design of primers to amplify bisulfite-treated DNA templates, necessary to determine genomic DNA methylation profiles. Indeed, the number of studies on bisulfite-treated templates exponentially increases as determining DNA methylation becomes more important in the diagnosis of cancers. Bisulfite-treated DNA is difficult to amplify since undesired PCR products are often amplified due to the increased sequence redundancy after the chemical conversion. In order to increase the efficiency of PCR primer-design, we have developed BiSearch web server, an online primer-design tool for both bisulfite-treated and native DNA templates.

**Results:**

The web tool is composed of a primer-design and an electronic PCR (ePCR) algorithm. The completely reformulated ePCR module detects potential mispriming sites as well as undesired PCR products on both cDNA and native or bisulfite-treated genomic DNA libraries. Due to the new algorithm of the current version, the ePCR module became approximately hundred times faster than the previous one and gave the best performance when compared to other web based tools. This high-speed ePCR analysis made possible the development of the new option of high-throughput primer screening. BiSearch web server can be used for academic researchers at the  site.

**Conclusion:**

BiSearch web server is a useful tool for primer-design for any DNA template and especially for bisulfite-treated genomes. The ePCR tool for fast detection of mispriming sites and alternative PCR products in cDNA libraries and native or bisulfite-treated genomes are the unique features of the new version of BiSearch software.

## Background

Polymerase chain reaction (PCR) is a widely used technique both in clinical diagnosis and research applications. The success of a PCR reaction is essentially determined by the oligonucleotides used as primers for the amplification of the target sequence. Although a large variety of primer-design softwares exist, some aspects of the design are rarely considered by these programs. For example only very few programs can design primers to amplify bisulfite treated DNA [1,2]. This is surprising since the number of studies including PCR on bisulfite treated DNA templates is exponentially growing. Bisulfite treated DNA is used to determine CpG methylation profile [3,4]. Methylation of the mammalian DNA influences gene expression regulation, cell division and chromosome stability [5]. Aberrant DNA methylation profiles are generally observed in pathological processes, such as cancerogenesis [6]. Bisulfite genomic PCR based diagnostic tools were developed to detect these aberrant methylation profiles [6]. The principle of the bisulfite treatment is the specific chemical conversion of non-methylated cytosines to uracils, while methyl-cytosines remain unconverted. These templates are then PCR amplified and uracils and methyl-cytosines are replaced in the product by thymines and cytosines, respectively. The amplification of a bisulfite treated sequence is a difficult task as the treated DNA sequences are characterized by a very high percentage of T, leading to low efficiency of amplification and high frequency of undesired PCR products. Indeed, amplification of undesired PCR products can seriously alter the efficiency of a reaction [7-9]. Surprisingly, another rarely addressed aspect of primer-design is the post-design in silico screen for mispriming sites and undesired PCR products (electronic PCR or ePCR). We have created BiSearch [8], a primer-design software, which considers both the above mentioned aspects of primer-design. After a complete upgrading of the software, here we describe the second version of BiSearch. The current version is composed of a primer-design and an ePCR module. The primer-design module is applicable to a wide variety of templates and was supplemented with new parameters used specifically for bisulfite treated DNA. The ePCR module can test primers for mispriming sites and misamplification products both in native or bisulfite treated genomic libraries. BiSearch can recognize imperfect matches as well, which are likely to anneal and cause potentially undesired PCR product amplification. The new version was also supplemented with eight cDNA libraries. The search is carried out with a completely new algorithm and became hundred times faster in the current program than it was in the previous version and is currently the fastest on the web. The rapidity of the ePCR search allowed the development of a new option of high-throughput primer screening, necessary in some applications.

## Implementation

BiSearch web server is fast and user friendly due to the combination of various techniques and programming languages. The BiSearch software is composed of two parts: the core applications and the web interface. The main programs (primer-design and ePCR) are written in C and C++ to ensure rapidity. For primer design we coded the algorithm proposed by Kämpke et. al [10] with some modification described previously [8]. The ePCR tool contains two programs. *Indexer *was developed for indexing databases and generating the appropriate hash matrices. *Indexer *is run only once for each new assembly of a database. The second part of the ePCR tool is the *fpcr *program. This program can take one or more primer(s) or primer pair(s) as input. First, it generates all oligonucleotides, which differ from the query sequence according to the mismatch string (see later). Then these oligonucleotides are sorted according to their hash index, and genomic localizations are given by the linked list designated by the appropriate hash matrix element. Potential PCR products are predicted by the further analysis of the generated genomic hits. In this step, the full-length primer sequences are taken into account.

The web interface of BiSearch is written in PHP. The main programs communicate by the PHP scripts via CGI. The different options and modules of BiSearch web server are structured by the main menu. A dynamic help, generated by javascript facilitates the use of the software.

## Results and discussion

### Primer design

Primers can be designed for native or bisulfite treated DNA sequences by the BiSearch web server. The software also proposes primer-design for methylation specific PCR (MSP) [11], when qualitative analysis of DNA methylation is needed after bisulfite treatment.

Due to the bisulfite treatment of DNA, the originally double-stranded, self-complementary DNA molecules loose the complementarity of the two strands. Therefore the original sense and antisense strands need separate amplification. Accordingly, BiSearch proposes to design primers either for the amplification of bisulfite treated sense or antisense strand. In both cases the bisulfite conversion is carried out by the software on the native input sequence. This allows the consideration during primer-design both the methylated and the unmethylated state of each CpG dinucleotide.

**Figure 1:**
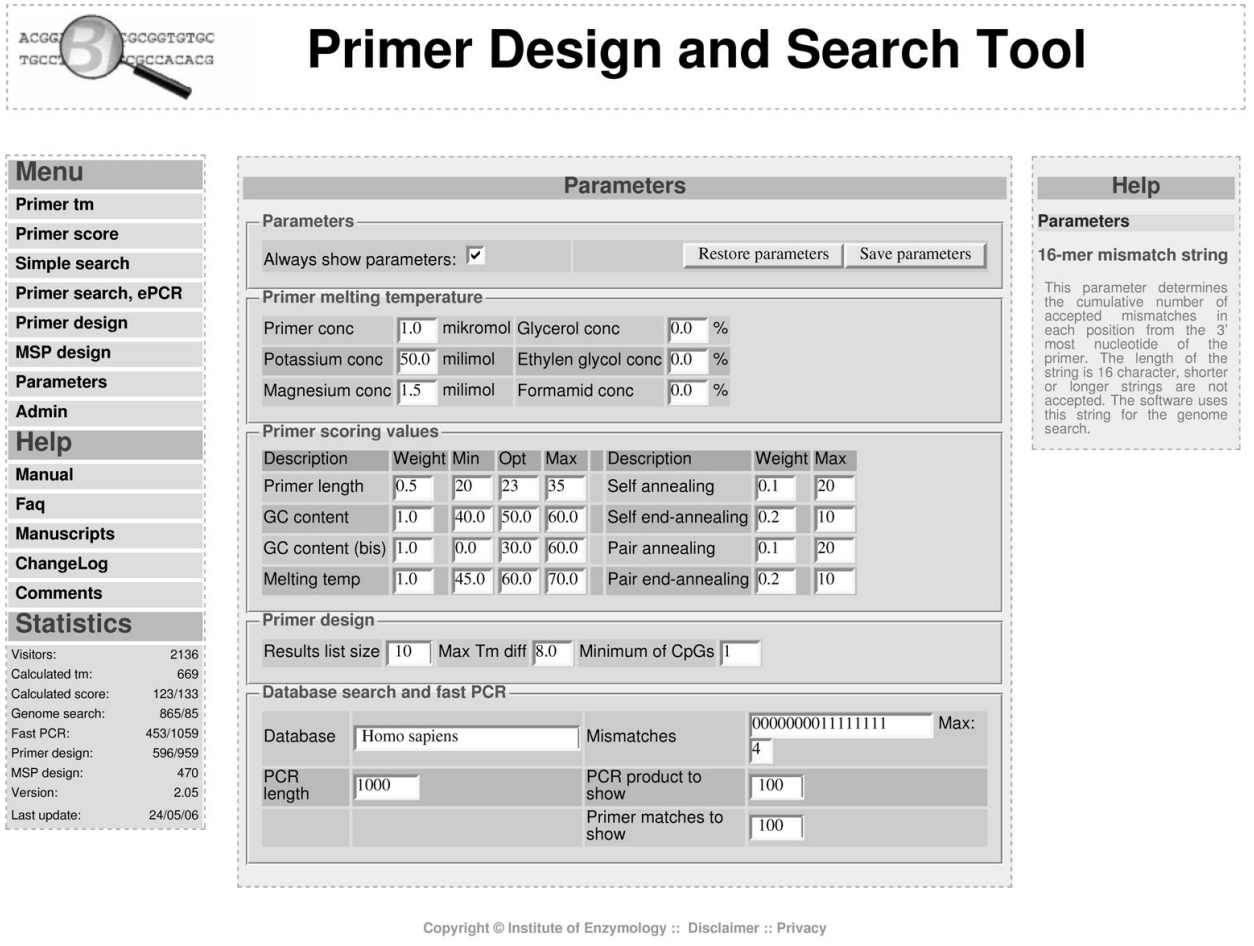
**Adjustable parameters of BiSearch**. Screenshot of adjustable parameters of BiSearch.

The following general parameters are considered for primer-design: self and pair (end) annealing of the primers, their melting temperature (Tm), GC content, length and maximum acceptable Tm difference between the forward and reverse primers. Although default parameters are given, each parameter used for primer-design (or ePCR) can be changed (see Figure 1). The software calculates the Tm with the nearest neighbor method, according to the published equations [12].

Some specific parameters are used only when the template is bisulfite treated. First, GC content in a bisulfite treated sequence is lower than in native sequences. Therefore, to make primer-design easier we have modified the default range of acceptable GC content for primer-design on bisulfite treated template. Second, since the objective of bisulfite treatment is to determine the DNA methylation profile of the target sequence, it was logical to include a minimal number of CpG dinucleotides in the PCR product, necessary for primer design. This can be determined by the user. Finally, the primers proposed by the software may include CpG dinucleotides of the original sequence. These primers are degenerate since at the corresponding site either a C or a T is incorporated. These degenerate primers are characterized by two (or more) different Tms according to the number and the methylation state of the CpG dinucleotides. The maximal acceptable Tm difference between the methylated and unmethylated primers can be set by the user.

BiSearch software proposes only significantly different primer pairs. Two primer pairs are significantly different from each other if the location of either the left or the right primer's 5' or 3' end has at least 3 base difference relative to the corresponding primer. The advantage of this approach is the possibility of the setting up of alternative PCRs to amplify the same template. This may become necessary when the best primer pair proposed by the software turns out to have a great number of mispriming sites and potential misamplification product(s) after the analysis by the ePCR tool (see below).

In case of primer-design for methylation specific PCR (MSP), the software calculates the Tm values for both a fully converted (each C to T) and a partially non-converted template (each C to T except for CpGs). In contrast to the "classic" bisulfite PCR, where the objective is the unbiased amplification of both methylated and unmethylated alleles, here the aim is the specific amplification of the methylated template. Therefore the software searches primers with high Tm difference. To increase the specificity for the methylated template, only those primer pairs are accepted where at least one of the primers has a(n unconverted) C at the 3' end.

**Figure 2:**
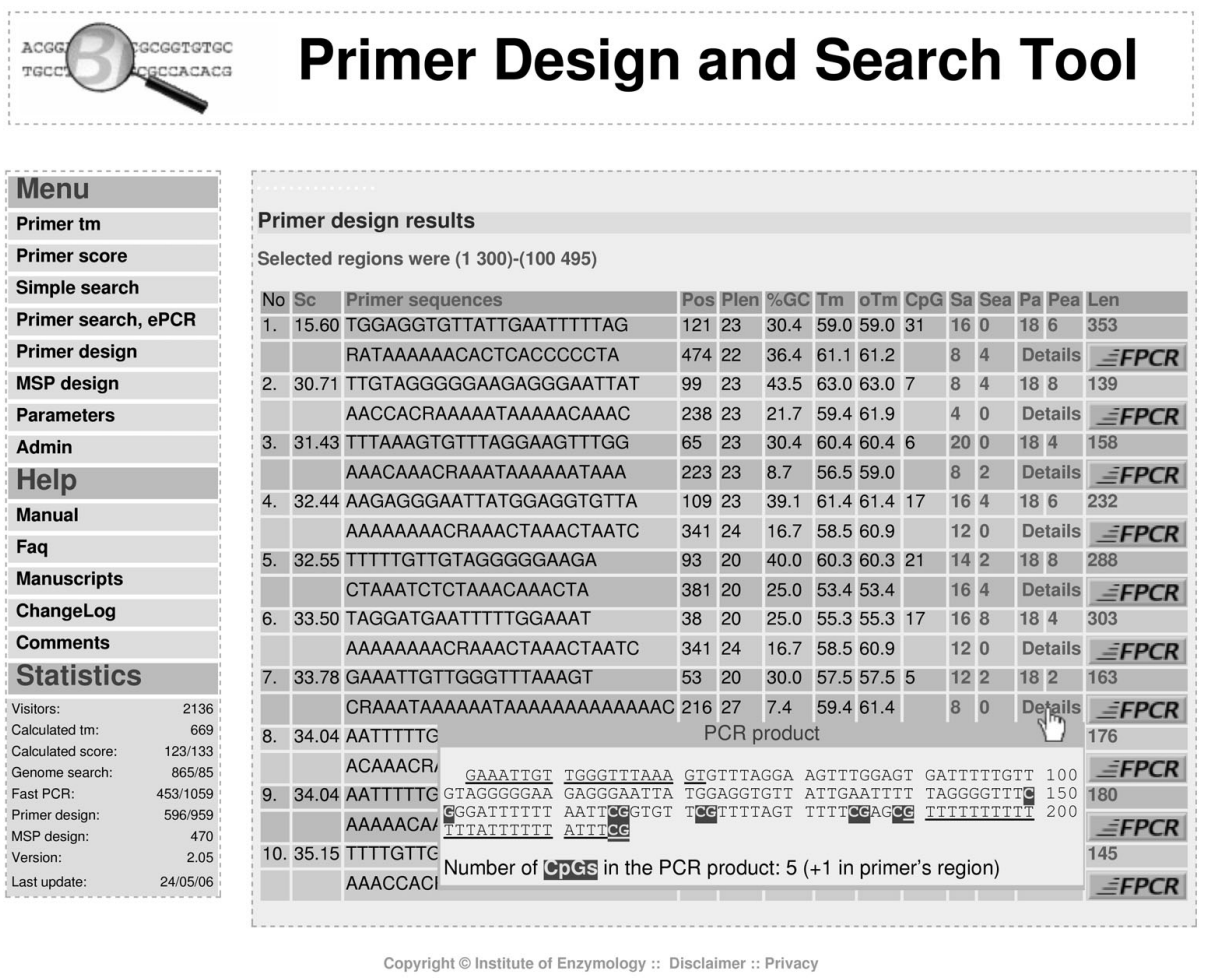
**Results of primer-design**. Screenshot of primer-design result page.

The score of primer pairs is calculated as described in [10]. Briefly, each parameter has a minimum, maximum and an optimum value determined by the user. Primers are considered when the value of all the parameters fall in the predefined range. The parameters are also characterized by a weight used for scoring. The score of a primer pair equals the simple weighted sum of the deviations from the optimum. Designed primer pairs are ranked in a table according to their scores where lower score means better primers (Figure 2). The table also includes the calculated values for the different parameters. The PCR amplicon is shown with the primers underlined and the CpG dinucleotides highlighted. The FPCR (fast PCR) button serves to start the post-design electronic PCR analysis.

### Post-design electronic PCR tool

Contaminating PCR products can seriously alter the efficiency of an amplification reaction. Unfortunately, all PCR templates (cDNA, native or bisulfite treated genomic DNA, etc.) can serve as mispriming site for primers leading eventually to undesired PCR amplicons. Selected primers can frequently bind to similar or identical sequences other than targeted in complex templates such as mammalian genomic DNA. Due to bisulfite treatment the complementarity of the strands is lost and high sequence redundancy is typical because of the T-richness after the conversion of Cs. Here, mispriming and misamplification happen much more often. This illustrates that post-design primer analysis for mispriming sites and more importantly for undesired PCR products is crucially important. The unique ePCR functions of BiSearch were created to meet these needs.

The ePCR tool in this new version of BiSearch is based on hashing of various template DNA libraries using 16 base long oligonucleotide sequences instead of direct string search. The principle of hashing technique is the initial identification of locations of all n-mer oligos (in our case 16-mers) in the databases to be analyzed. The hash tables are generated for cDNA and both bisulfite converted and native genome sequences. Three hash tables are generated for each genome, one for the original sequence and two for the bisulfite converted non-complementary DNA strands. The hash tables are splitted up into smaller parts to avoid large files and to speed up search. Each occurrence of the analyzed primer sequence in the selected template DNA library is collected as a result of an ePCR search.

However, mispriming may also occur (with lower affinity) at sequences, which are almost identical to the primer but harbor some mismatches. Since the hashing technique allows the identification of only exact matches, we developed a new algorithm to identify primer-binding sites with mismatches. The software generates all possible primer sequences differing from the original primer sequence as defined by a "mismatch string". The mismatch string is a 16 character long string, where each position represents the corresponding nucleotide from the 3' end of the primer. The string is composed of numbers indicating the maximum allowable cumulative number of mismatches in the primer from the 3' end to the specific position. The default mismatch string is (0000000011111111), which means no mismatch is acceptable within the first eight nucleotides from the 3' end of the primer, while sequences with one mismatch between nucleotides 9–16 from the 3' end are identified as binding sites for the oligo. This approach identifies hits with more stringent 3' than 5' end similarity to the primer sequence. These hits may serve as templates for the amplification of undesired PCR products with higher probability than sequences with higher overall but lower 3' end similarity.

After the collection of hits, the software identifies the potential PCR products by the position codes in the template DNA library. Two hits are considered as primer binding sites leading to PCR amplification if they are well oriented and are not more distanced than 1 kb (the default parameter can be modified by the user). Full-length primer sequences are used for the identification of potential PCR products and sequences having more mismatches than allowed for the entire oligonucleotide are discarded. Four mammalian genomes (human, chimpanzee, mouse and rat) were downloaded from Ensembl ftp site [13] and were indexed. Hash tables for the cDNA libraries of these species were also prepared in addition to bovine, zebrafish, fruit fly and C. elegans cDNA sequences. Hash tables for further genomic and cDNA sequences can be prepared upon request.

The output format of the results of a simple genome search with a single oligonucleotide is the listing of the genomic hits with their precise location and an alignment between the primer and the hit sequences. If the search is carried out on a bisulfite treated genome the results are separately shown for the (original) sense and antisense strands. The primer genome search report starts with the total number and the complete sequence of the potential PCR products. The primers are color-coded in the PCR products and mismatches are highlighted. The PCR data are followed by the (mis)priming sites. Results from bisulfite treated templates are given first for the original sense then for the antisense strands.

In general, search time of binding sites of a single oligonucleotide with the default parameters in a non-converted genome is approximately 20 seconds. The same search on the bisulfite treated genomes takes approximately 30 seconds, which is approximately hundred times faster than it was in the previous version of BiSearch. The search time depends on the number of generated mismatch strings. However, the main time consuming step in the algorithm is to find the appropriate position list in the hash tables, therefore strings having position lists close together can be found more quickly. Thus sorting the primers according to their hash index speeds up the search. Moreover, simultaneous ePCR analysis of several primers is faster than separate search with each sequence. In conclusion, the simultaneous analysis of multiple primer pairs and their initial sorting according to their position lists contributed to that BiSearch became a high-throughput online primer screening ePCR tool.

Both the efficiency and the rapidity of the ePCR module were tested and compared to other web tools. BiSearch is the only available online tool to our knowledge to predict potential PCR amplification products of a primer pair on a bisulfite treated template. Mispriming sites of a single oligonucleotide can be also predicted by methBLAST, a derivative of the BLAST software [14]. In contrast to BiSearch, methBLAST software readily detects sequences with mismatches corresponding to the 3' end of the primer, while a sequence with a single mismatch corresponding to the middle of the primer is hardly recognized. Thus, it is more likely that the hits identified by BiSearch can serve to initiate elongation by Taq polymerase than the hits of methBLAST.

The ePCR module of BiSearch was also compared to other online tools available for native sequences. The currently available fastest program for electronic PCR on native genomic DNA is the GenomeTester program part of the GenomeMasker software package described recently [15]. Groups of randomly selected 100 human primer pairs were used from the RTprimerDB [16] to compare GenomeTester and BiSearch. The search time with the default parameters of BiSearch (allowing mismatches) was consequently three times shorter than that observed with the online available GenomeTester software, which detects only identical matches.

We have also compared BiSearch to two other previously developed ePCR softwares, VPCR [17] and SPCR [18]. In all our tests BiSearch showed the best performance, since it was not only very fast but also predicted almost all real PCR products, while no false undesired amplification was suggested. This is due to the fact that BiSearch is the only software out of the three, which uses real text search algorithm to identify the potential PCR products.

A new feature of BiSearch is the possibility to analyze primer pairs on cDNA libraries. This option was also tested on the same randomly selected 100 primer pairs. Not surprisingly the search on the cDNA library revealed potential PCR products, which remained undetected by the test on the genomic templates. Furthermore, in some cases the same primer pair detected several different PCR products from the cDNA library corresponding to potential alternative splicing products. Therefore, we suggest to test each primer pair designed to amplify a cDNA on both the corresponding cDNA library (for alternatively spliced products) and the native genomic library (for undesired PCR products from a potential genomic DNA contamination).

## Conclusion

In conclusion, BiSearch web server is a useful tool for primer-design for any DNA template and especially for bisulfite treated sequences. The ePCR tool for detection of mispriming sites and alternative PCR products in cDNA libraries and native or bisulfite treated genomes are the unique features of the new version of BiSearch software. The major differences between BiSearch and the other softwares are that the ePCR tool presented here can search: i) in both cDNA and genomic libraries (currently 12 different native libraries); ii) in bisulfite treated genomic libraries (currently four bisulfite treated libraries); iii) for hits identical to the query sequence or harboring mismatches with high probability of 3' end annealing and iv) faster than any similar online available software. These improvements of the BiSearch software make it a highly performant and versatile primer-design web tool.

## Competing interests

The author(s) declare that they have no competing interests.

## Availability and requirements

• Project name: BiSearch: Primer Design and Search Tool

• Project home page: 

• Operating system(s): Linux on server side, platform independent on client sides

• Programming language: C, C++, JavaScript, PHP, HTML

• License: free for academic users

• Any restrictions to use by non-academics: licence needed

## Authors' contributions

GET developed and implemented the tool and drafted the manuscript. TA participated in design and preparation of manuscript. AV offered technical help during the development. IS and AV supervised the project. All the authors read and approved the manuscript.
